# Bibliometric analysis of research trends on the epiretinal membrane since 2000

**DOI:** 10.3389/abp.2026.15244

**Published:** 2026-02-12

**Authors:** ShuHan Zeng, Rui Wang, Jie Chen, Yue He

**Affiliations:** 1 Department of Ophthalmology, The Affiliated Hospital of Southwest Medical University, Luzhou, Sichuan, China; 2 Department of Rheumatology and Immunology, The Affiliated Hospital of Southwest Medical University, Luzhou, Sichuan, China

**Keywords:** bibliometric analysis, bibliometrix, citespace, epiretinal membrane, VOSviewer

## Abstract

Epiretinal membrane (ERM) is an important retinal disorder, yet its global research landscape has not been systematically evaluated. This study conducted a comprehensive bibliometric analysis of ERM-related publications indexed in the Web of Science Core Collection. Data were analyzed using CiteSpace, bibliometrix, VOSviewer, and the bibliometrix R package to assess publication trends, international collaboration, influential journals, and evolving research topics. A total of 1,059 articles published between 2000 and 2024 were included. The annual number of publications showed a steady increase, with the United States and other developed countries contributing the majority of research output. Journal analysis identified Retina-The Journal of Retinal and Vitreous Diseases as the leading outlet in terms of productivity. Keyword co-occurrence and co-citation analyses revealed that current research hotspots involve epidemiology, molecular mechanisms, imaging biomarkers associated with surgical outcomes, and the emerging use of artificial intelligence in diagnosis and prognosis prediction. These findings provide an integrated overview of ERM research development, highlight major thematic shifts, and offer valuable guidance for future investigations in this field.

## Introduction

The epiretinal membrane (ERM) is a fibrocellular proliferation occurring on the inner surface of the retina and is commonly associated with visual distortion and decreased central vision (Kanda et al., 2019; Holzwarth et al., 2024). The clinical diagnosis of ERM has evolved substantially with the introduction of optical coherence tomography (OCT), which enables high-resolution visualization of retinal microstructure. Advances in spectral-domain OCT (SD-OCT) have further facilitated the development of imaging-based classification systems that improve diagnostic consistency and understanding of disease severity (Fung et al., 2021; Stevenson et al., 2016; Dupas et al., 2015).

ERM is a common retinal disease in clinic, and its incidence rate is positively correlated with age (Matoba and Morizane, 2021; Kawasaki et al., 2009). It may also occur secondary to various retinal diseases or intraocular surgeries, imposing a substantial burden on visual function and quality of life. Although pars plana vitrectomy remains the standard treatment, uncertainties remain regarding optimal surgical timing, prognostic biomarkers, and underlying pathogenic mechanisms (Miyazaki et al., 2003; Matoba and Morizane, 2024). Consequently, ERM has attracted growing scientific and clinical interest, leading to a notable rise in publications addressing its epidemiology, molecular features, diagnostic technologies, and treatment strategies.

Despite this expanding body of literature, no comprehensive bibliometric study has yet analyzed global research trends, influential contributors, thematic evolution, and emerging hotspots in ERM research. Bibliometrics applies quantitative statistical methods to evaluate the structure and dynamics of scientific output, providing a systematic means to map knowledge development and identify future research directions (Thompson and Walker, 2015; Ninkov et al., 2022).

To address this gap, the present study performed a bibliometric analysis of ERM-related publications indexed in the Web of Science Core Collection from 2000 to 2024. Using CiteSpace, VOSviewer, and the bibliometrix R package, we analyzed publication patterns, country and institutional contributions, journal characteristics, influential references, and evolving research themes, with the aim of providing an integrated overview of ERM research and guidance for future investigations.

## Materials and methods

We conducted a comprehensive literature search in the Web of Science Core Collection database (WOSCC) on September 24, 2024 using the following search terms: TS=(“epiretinal membrane”) OR (“epidermal membrane”) OR (“macular pucker”) OR (“cellophane maculopathy”). The inclusion criteria were: (1) publication period from 1 January 2000 to 24 September 2024; (2) document types restricted to article and review; and (3) publications written in English. The search initially retrieved 3,348 records. Two independent researchers screened the titles and abstracts to exclude irrelevant papers, and any disagreements were resolved by a third reviewer. A total of 1,061 publications met the eligibility criteria and were included in the analysis.

Bibliometric analyses were performed using VOSviewer 1.6.20, CiteSpace.6.3, and the R package Bibliometrix 4.2.1. VOSviewer was used to construct co-occurrence and co-authorship networks reflecting collaborations among countries, institutions, journals, and keywords (van Eck and Waltman, 2010). CiteSpace was applied to identify emerging trends through timeline visualizations and keyword burst detection (Synnestvedt et al., 2005). Bibliometrix was used to generate descriptive publication indicators, including annual output, most productive countries, institutions, journals, and highly cited references (Aria and Cuccurullo, 2017). All analyses were descriptive in nature, and no inferential statistical tests were required or applied, consistent with standard bibliometric methodology.

## Results

### Overall results of the publication search

**FIGURE 1:**
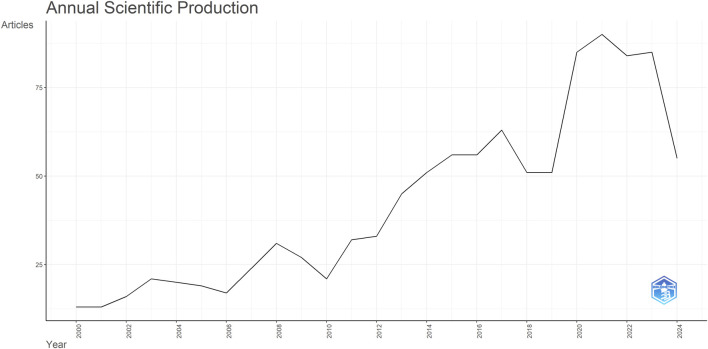
Trends in annual publications on epiretinal membrane (ERM) research from 2000 to 2024.

A total of 1,061 ERM-related publications were initially retrieved from WOSCC. After duplicate removal using Citespace, 1,059 publications were included in the bibliometric analysis. These studies were authored by 4,107 researchers affiliated with 53 countries/regions and were published across 141 journals, comprising 1,023 research articles and 36 review articles. As shown in Figure 1, the annual number of ERM publications exhibited a steady upward trajectory from 2000 to 2024, indicating growing scientific interest and sustained research activity in this field.

### Analysis of countries/regions

**FIGURE 2:**
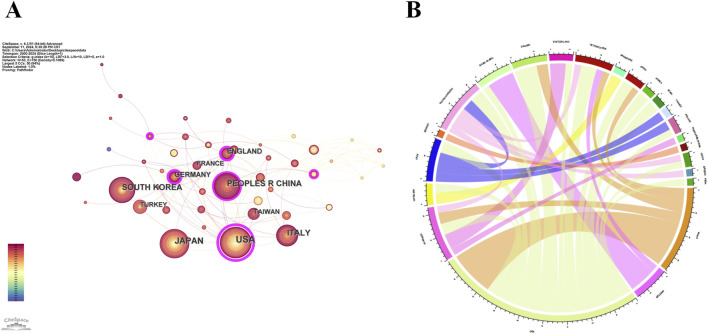
International collaboration network of countries/regions involved in ERM research. **(A)** Co-authorship network showing publication volume and collaborative links. **(B)** Chord diagram illustrating the proportion of publications contributed by each country/region.

The 1,059 publications originated from 53 countries/regions. Figure 2 illustrates the global distribution and collaboration network. In the co-authorship map (Figure 2A), each node represents a country/region, with node size proportional to publication volume and link thickness indicating collaboration strength. In the chord diagram (Figure 2B), the United States accounts for nearly one-quarter of all publications, reflecting its leading role in ERM research. The United States demonstrated the broadest international collaborations, with China being its most frequent partner. Japan showed strong bilateral collaborations with both the United States and China. Table 1 lists the top 10 most productive countries/regions. The United States ranked first (188), followed by Japan (172) and China (124). Among them, publications from Japan accumulated the highest total citations (5,258), highlighting their substantial academic influence.

**TABLE 1:** The top 10 most productive countries and regions.

Country/Region	Publications	Citations	Centrality
USA	188	4847	0.7
Japan	172	5258	0.01
Peoples R China	124	1691	0.12
South Korea	115	1976	0
Italy	94	1653	0.05
England	58	1424	0.26
Germany	55	1424	0.27
Taiwan	43	354	0
France	40	1094	0
Turkey	36	300	0

### Analysis of authors

A total of 4,107 researchers contributed to the 1,059 ERM-related publications included in this study, reflecting a broad and diverse authorship base within the field. To characterize collaboration dynamics, a group-level co-authorship network was constructed. The network analysis revealed a multi-cluster structure, in which researchers tended to collaborate within relatively cohesive local groups, whereas cross-cluster or broader international collaborations remained limited. These findings suggest that while ERM research activity is substantial, author collaboration patterns are still fragmented, indicating potential opportunities for strengthening interdisciplinary and cross-institutional cooperation in the future.

### Analysis of institutions

Tables 2, 3 summarize the institutions with the highest publication output and the highest betweenness centrality. The results showed that Harvard University (USA) produced the largest number of publications (22), followed by Harvard Medical School (USA) and Capital Medical University (China). It is worth mentioning that due to Harvard Medical School being affiliated with Harvard University, there is a certain degree of overlap in the publication of articles between the two institutions.

**TABLE 2:** Top 10 institutions with highest productivity.

Rank	Institution	City	Country/Region	Publications
1	Harvard University	Boston	USA	22
2	Harvard Medical School	Boston	USA	19
3	Capital Medical University	Beijing	China	17
4	University College London	London	UK	16
5	University of California System	Multiple campuses	USA	14
6	Assistance Publique Hopitaux Paris (APHP)	Paris	France	14
7	Seoul National University (SNU)	Seoul	South Korea	14
8	University of London	London	UK	13
9	Moorfields Eye Hospital NHS Foundation Trust	London	UK	13
10	National Taiwan University Hospital	Taipei	Taiwan	13

**TABLE 3:** Top 10 institutions with the highest centrality.

Rank	Institution	City	Country/Region	Centrality
1	University of California System	Multiple campuses	USA	0.11
2	Harvard Medical School	Boston	USA	0.07
3	Academic Medical Center Amsterdam	Amsterdam	Netherlands	0.07
4	University College London	London	UK	0.05
5	Azienda Ospedaliero Universitaria Careggi	Florence	ITALY	0.05
6	Egyptian Knowledge Bank (EKB)	Cairo	EGYPT	0.05
7	Harvard University	Boston	USA	0.04
8	University of Florence	Florence	ITALY	0.04
9	CHU de Nantes	Nantes	FRANCE	0.04
10	Capital Medical University	Beijing	CHINA	0.03

In the centrality analysis, which reflects the role of an institution in bridging collaborative networks, the top-ranked institutions were predominantly based in the USA. This indicates that USA institutions not only contribute substantially to research output but also occupy structurally influential positions in the global ERM collaboration network.

### Analysis of journals

The 1,059 publications appeared in 440 different journals. Table 4 lists the top 10 most productive journals, with Retina-The Journal of Retinal and Vitreous Diseases publishing the highest number of articles (181), followed by Graefes Archive for Clinical and Experiential Ophthalmology (86) and Inestigative Ophthalmology&Visual Science (56). Across all publications, a total of 13,275 references were cited, spanning 2,089 journals. The top 10 most frequently cited journals are shown in Table 5, with American Journal of Ophthalmology, Ophthalmology, and Retina ranking as the three leading sources of influential ERM literature.

**TABLE 4:** The top 10 most productive journals.

Source	NP	TC	JCR	H-index	IF
Retina-The Journal of Retinal And Vitreous Diseases	181	4221	Q2	36	3.3
Graefes Archive For Clinical And Experimental Ophthalmology	86	1894	Q2	24	3.5
Investigative Ophthalmology & Visual Science	56	2147	Q1	29	4.4
American Journal of Ophthalmology	53	3203	Q1	32	4.2
British Journal of Ophthalmology	46	1870	Q1	26	4.1
Eye	38	953	Q1	17	3.9
Bmc Ophthalmology	33	154	Q3	8	2
Ophthalmology	29	2049	Q1	24	13.7
Ophthalmologica	28	392	Q2	11	2.6
European Journal of Ophthalmology	27	190	Q4	8	1.7

**TABLE 5:** The top 10 most cited journals.

Source	Articles
American Journal of Ophthalmology	3,585
Ophthalmology	3,443
Retina-The Journal of Retinal And Vitreous Diseases	3,349
Investigative Ophthalmology & Visual Science	2,814
Graefes Archive For Clinical And Experimental Ophthalmology	1,689
British Journal of Ophthalmology	1,670
Arch Ophthalmol-Chic	909
Eye	811
Acta Ophthalmol	447
Plos one	399

**FIGURE 3:**
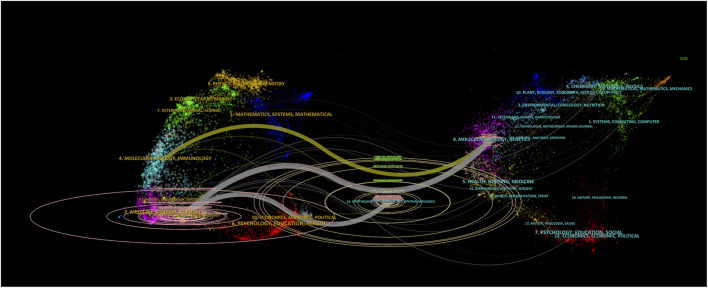
Dual-map overlay of journals, showing citation pathways between citing and cited journal clusters.

The dual-map overlay (Figure 3) illustrates the disciplinary distribution of citing and cited journals. In this visualization, the left side represents the disciplinary domains of the citing literature, while the right side shows the domains of the cited literature. The major citation pathways—indicated by pink and yellow trajectories—demonstrate that ERM studies are primarily published in journals associated with “Molecular, Biology, Imminology” as well as “Neulogy, Sports, and Ophthalmology”, and these works predominantly cite literature from “Molecular, Biology and Immunology” and Ophthalmology-related disciplines. This reflects the interdisciplinary nature of ERM research, bridging molecular mechanisms with clinical ophthalmology.

### Analysis of reference

The 1,059 publications cited a total of 13,275 references. Local Citation Score (LCS), which measures the citation frequency of a reference within the retrieved dataset, was used to identify the most influential works in ERM research. The top 10 references ranked by LCS are presented in Table 6. The highest-ranked publication was “Prevalence and Associations of Epiretinal Membranes: The Blue Mountains Eye Study”, followed by “Insights Into Epiretinal Membrane: Presence of Ectopic Inner Foveal Layers and a New Optical Coherence Tomography Staging Scheme” and “Five Year Cumulative Evidence and Progress of Epithelial Membranes: The Blue Mountains Eye Study”. These studies collectively represent foundational contributions in epidemiology, OCT-based phenotyping, and disease progression.

**TABLE 6:** The top 10 references with highest LCS.

Title	IF	Year	Journal	Citations
Prevalence and associations of epiretinal membranes. The blue mountains eye study, Australia	13.1	1997	Ophthalmology	194
Insights into epiretinal membranes: presence of ectopic inner foveal layers and a new optical coherence tomography staging scheme	4.5	2017	Am J Ophthalmol	161
Five-year cumulative incidence and progression of epiretinal membranes: the Blue Mountains Eye Study	13.1	2003	Ophthalmology	136
Optical coherence tomography of idiopathic macular epiretinal membranes before and after surgery	4.1	2000	Am J Ophthalmol	123
Associations between macular findings by optical coherence tomography and visual outcomes after epiretinal membrane removal	4.1	2009	Am J Ophthalmol	122
Idiopathic epiretinal membrane	3.8	2014	Retina-J Ret Vit Dis	111
The epidemiology of epiretinal membranes	—	1994	Trans Am Ophthalmol Soc	109
Characterization of epiretinal membranes using optical coherence tomography	7.7	1996	Ophthalmology	105
Prevalence and associations of epiretinal membranes in the visual impairment project	4.1	2005	Am J Ophthalmol	102
Correlation of visual recovery with presence of photoreceptor inner/outer segment junction in optical coherence images after epiretinal membrane surgery	3.6	2009	Brit J Ophthalmol	102

**FIGURE 4:**
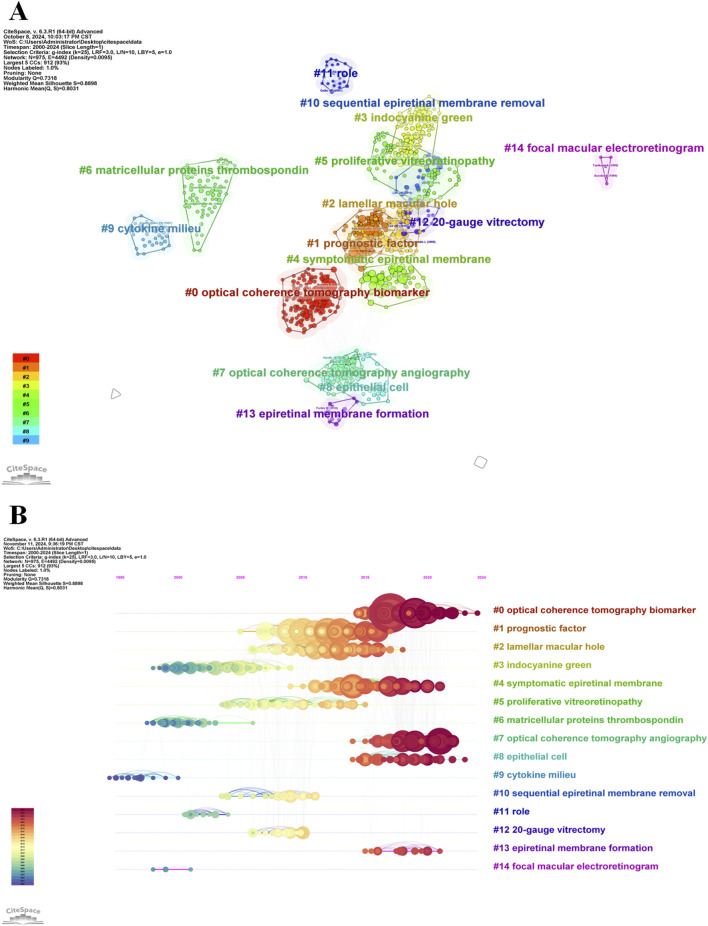
Co-cited reference analysis of ERM-related literature. **(A)** Cluster analysis of co-cited references. **(B)** Timeline distribution of the most active 15 clusters.

To further explore the intellectual structure of the field, a reference co-citation analysis was performed. Co-citation occurs when two references are cited together in subsequent publications, reflecting a conceptual relationship between them. As shown in Figure 4A, the co-citation network was partitioned into 64 clusters. The modularity Q value was 0.7318, and the average silhouette score reached 0.8898, indicating a well-structured and highly consistent clustering solution (Chen et al., 2012).

Figure 4B displays the temporal evolution of the major clusters, revealing clear shifts in research focus over time. Early clusters primarily centered on topics such as cytochrome milieu, mitochondrial proteins, and macroscopic electroretinogram techniques. In contrast, more recent clusters highlight emerging interests in optical coherence tomography biomarkers, optical coherence tomography angiography, and epithelial cell–related mechanisms. These trends reflect the transition of ERM research from foundational pathophysiological studies toward advanced imaging biomarkers and microstructural characterization.

### Keywords co-occurrence analysis

**FIGURE 5:**
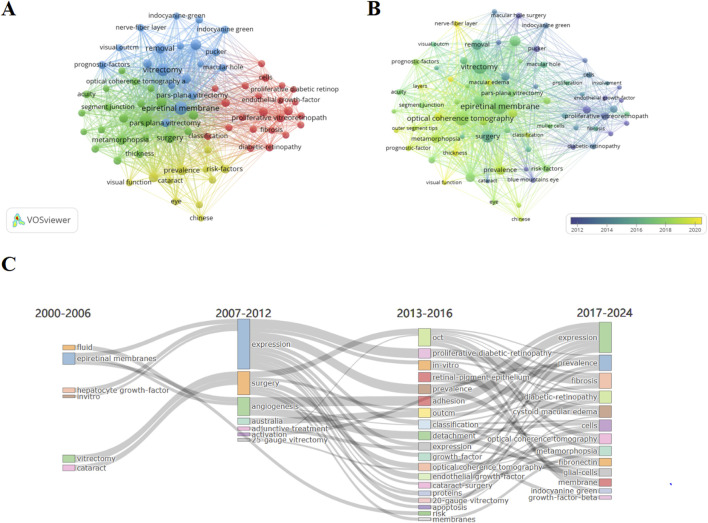
Keyword-based visual analyses of ERM research. **(A)** Keyword co-occurrence network generated by VOSviewer. **(B)** Overlay visualization showing the average publication year of keywords. **(C)** Sankey diagram illustrating the thematic evolution of ERM research over time.

A keyword co-occurrence analysis was performed using VOSviewer to identify major research themes and their temporal evolution within the ERM literature. A total of 74 keywords appeared at least 20 times and were included in the visualization. In the co-occurrence network, node size reflects the frequency of keyword occurrence, while the color gradient represents the average publication year (APY). As shown in Figure 5B, earlier topics (purple nodes) were associated with general clinical features, whereas more recent topics (yellow nodes) highlight emerging interests such as “nerve fiber layer” and “visual functions,” indicating a shift toward microstructural and functional assessments.

To further characterize the evolution of thematic areas, a thematic evolution map generated using the bibliometrix package is presented in Figure 5C. The results demonstrate a progressive expansion in the diversity of research keywords over time. New terms—including “fibrosis”, “diabetic retinopathy”, and “cystoid macular edema”—have appeared in recent years, suggesting broader clinical and mechanistic investigations. The keyword “expression”, which was prominent between 2007 and 2012, re-emerged after 2017, reflecting ongoing interest in molecular pathways associated with ERM.

**FIGURE 6:**
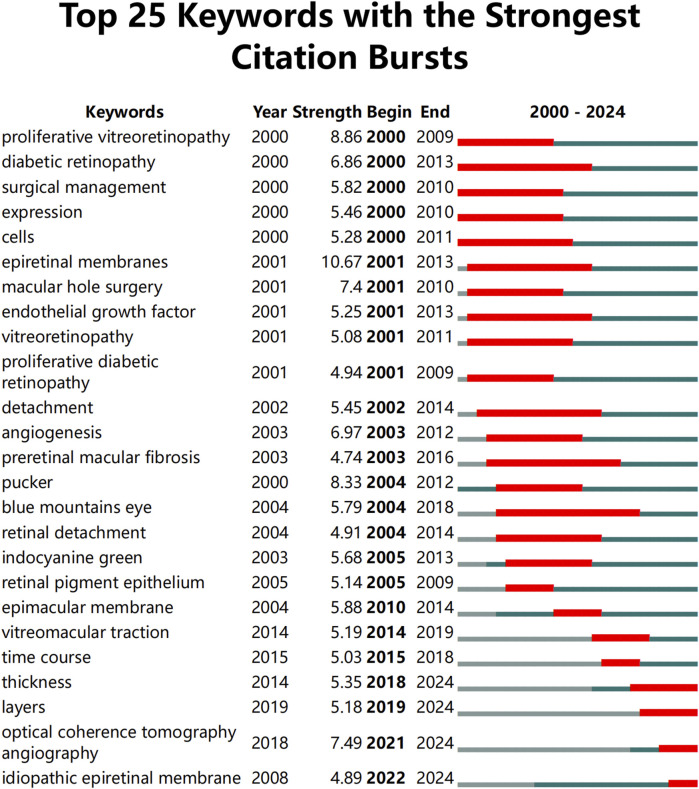
The top 25 keywords with the highest citation burst from 2000 to 2024.

Keyword burst detection conducted in Citespace (Figure 6) identified 44 burst terms, representing topics that gained significant attention during specific time periods. Early bursts centered on “proliferative vitreoretinopathy”, whereas more recent bursts highlight growing interest in “idiopathic epiretinal membrane” and “optical coherence tomography angiography”.

Collectively, these findings illustrate the dynamic development of ERM research, transitioning from foundational pathological descriptions to advanced imaging biomarkers and mechanistic exploration.

## Discussion

This study provides the first comprehensive bibliometric and visualization analysis of global research on ERM over the past 2 decades. By examining publication trends, collaborative networks, influential institutions, journals, and research hotspots, our findings offer an objective overview of the evolution and current landscape of ERM research.

Our analysis revealed a steadily increasing annual publication trend, reaching its peak in 2021, indicating growing academic interest in ERM. Countries such as the United States, England, and Germany demonstrated the highest centrality in the global collaboration network. In bibliometric analysis, high centrality reflects strong academic influence and active international cooperation. The United States, in particular, holds a central position, likely due to its longstanding research strength and influential contributions to ERM-related diagnostic and surgical technologies. For instance, innovations such as the 25-gauge transconjunctival sutureless vitrectomy system introduced by Fujii et al. (2002) and advances in ultrahigh-resolution ophthalmic OCT developed by Drexler et al. (2003) have shaped current clinical and research practices. These landmark studies help explain the United States’ academic leadership in the field. The high centrality of the UK and Germany further reflects active participation in multi-country collaborations and widespread recognition of their research outputs.

At the institutional level, Harvard University (USA) had the highest number of publications, supported by its strong academic resources and access to large clinical datasets from Massachusetts Eye and Ear. Influential contributions include Aiello et al.’s research identifying TGF-β2–mediated mechanisms in ERM contraction (Kohno et al., 2009) and S. Pennock et al.’s early insights into anti-VEGF therapy for proliferative vitreoretinopathy (Pennock et al., 2013), both of which have been widely cited and have inspired subsequent studies (Nagar et al., 2020). Capital Medical University (China) ranked third globally in publication output, reflecting China’s increasing visibility in ERM research. Liu et al.’s OCTA-based evaluation of choroidal capillary perfusion in ERM patients demonstrates how recent work from emerging research centers contributes to clinical understanding and has gained rapid academic recognition (Yu et al., 2017).

For the publication status of journals, our aim was to provide researchers with useful guidance for literature retrieval and journal selection through a visual analysis of bibliometric indicators. RETINA has the highest number of publications, consistent with its focus on retinal diseases and its recognized academic influence in this field. RETINA also has the highest H-index among the journals analyzed. The H-index reflects the combined citation impact and productivity of a journal (Hirsch, 2005). Therefore, the high H-index of RETINA suggests that its published articles are both frequently cited and produced in substantial volume (Choudhri et al., 2015). In addition, ophthalmology owns the highest impact factor (IF), and three of the top ten most cited ERM-related publications were published in this journal. The journal with the highest citation count is American Journal of Ophthalmology. These complementary indicators—publication volume, H-index, impact factor, and citation counts—collectively illustrate the contribution and influence of journals in the ERM research landscape. Researchers may refer to these journals for recent advances in the pathogenesis, diagnosis, and treatment of epiretinal membrane.

Recent ERM studies have increasingly focused on pathogenesis, epidemiological characteristics, and surgical outcomes, which aligns well with the keyword co-occurrence clusters identified in our bibliometric analysis. Several molecular and population-based studies support these emerging directions. For example, investigations into cellular composition and molecular markers of ERM have contributed to foundational understanding of disease formation, while large population cohorts such as the Blue Mountains Eye Study and the Beaver Dam Eye Study have clarified epidemiological patterns, including age-related risk and ethnic variations in prevalence (Fraser-Bell et al., 2003; Meuer et al., 2015; Cheung et al., 2017; Kim et al., 2022; Ng et al., 2011). These findings are consistent with the thematic clusters observed in our analysis and illustrate the multidimensional development of ERM research.

Advances in ophthalmic imaging have led to the identification of more asymptomatic ERM cases, contributing to increased research interest in surgical outcomes and prognostic indicators—two major hotspots observed in this study. Although vitrectomy remains the only effective treatment, ongoing debate regarding optimal surgical techniques, such as inner limiting membrane (ILM) peeling, continues to stimulate clinical investigation (Schechet et al., 2017; Azuma et al., 2017; Zhang et al., 2023; Ducloyer et al., 2024; Chua et al., 2022). Recent work has increasingly focused on identifying imaging-based predictors of postoperative visual outcomes. For instance, Chou et al. (2024) reported a correlation between postoperative widening of temporal vascular angles and improvement in best corrected visual acuity (BCVA), while Shen et al. (2024) demonstrated that OCT-derived retinal microstructural parameters may help predict stereopsis after vitrectomy. These findings support the rising prominence of “biomarker” and “prognosis-related” keywords within our bibliometric clusters, reflecting the shift toward evidence-based and individualized surgical decision-making.

Artificial intelligence (AI) has emerged as a particularly dynamic research frontier, consistent with the burst keywords detected in our analysis. Deep learning models have been used to predict postoperative visual acuity with notable accuracy, as shown by Wen et al. (2023), and to synthesize postoperative OCT images, as demonstrated by Kim and Chin (2023) In addition to predicting surgical efficacy, AI-based diagnostic systems have also shown promising performance in ERM grading (Hung et al., 2023; Yan et al., 2024). Collectively, these studies underscore the increasing integration of AI into ERM diagnostics, prognostic modeling, and clinical decision-support systems, addressing both efficiency and resource-distribution challenges in ophthalmic care (Dong et al., 2022).

Despite its strengths, this study has several limitations. First, the analysis was restricted to WOSCC and English-language publications, which may have excluded relevant studies indexed elsewhere. Second, while bibliometric software objectively maps knowledge structures, it cannot fully represent the depth of scientific content within each topic due to space constraints. Finally, although two researchers independently screened publications, some subjectivity may remain.

In conclusion, this bibliometric analysis reveals the dynamic evolution of ERM research, identifies influential contributors and major publication venues, and highlights emerging themes such as imaging biomarkers, surgical prognostic indicators, and AI-assisted analysis. These insights may help guide researchers in identifying knowledge gaps, selecting potential collaborators, and recognizing future directions in ERM investigation.

## Data Availability

All the data analyzed in this study were obtained from publicly available academic databases: Web of Science Core Collection database (WOSCC). The website address is https://www.webofscience.com.
